# Diskriminierungssensible Sprache in der Forschung zu Migration und Gesundheit – eine Handreichung

**DOI:** 10.1007/s00103-022-03620-0

**Published:** 2022-11-21

**Authors:** Leman Bilgic, Navina Sarma, Anne-Kathrin M. Loer, Carmen Koschollek, Kayvan Bozorgmehr, Oliver Razum, Claudia Hövener, Katja Kajikhina

**Affiliations:** 1 Institute of Occupational Medicine, Charité-Universitätsmedizin Berlin, Corporate Member of Freie Universität Berlin and Humboldt Universität zu Berlin, Berlin, Deutschland; 2 Alice Salomon Hochschule Berlin, Berlin, Deutschland; 3 Abteilung für Infektionsepidemiologie, Robert Koch-Institut, Berlin, Deutschland; 4 Abteilung für Epidemiologie und Gesundheitsmonitoring, Robert Koch-Institut, Berlin, Deutschland; 5 AG Bevölkerungsmedizin und Versorgungsforschung, Fakultät für Gesundheitswissenschaften, Universität Bielefeld, Bielefeld, Deutschland; 6 AG Epidemiologie & International Public Health, Fakultät für Gesundheitswissenschaften, Universität Bielefeld, Bielefeld, Deutschland; 7 Forschungszentrum gesellschaftlicher Zusammenhalt (FGZ), Standort Bielefeld, Universität Bielefeld, Bielefeld, Deutschland

**Keywords:** Antidiskriminierung, Soziale Ungleichheit, Migration, Sprache, Public-Health-Forschung, Anti-discrimination, Social inequality, Migration, Language, Public health research

## Abstract

**Hintergrund:**

In der Public-Health-Forschung ist Migration als eine Determinante von Gesundheit zunehmend in den Fokus gerückt. Verantwortungsvolle Forschung in diesem Bereich setzt eine antidiskriminierende Vorgehensweise in der Durchführung, Berichterstattung und Ergebnisdissemination voraus. Ein diskriminierungssensibler Sprachgebrauch ist dabei ein zentrales Element. Handreichungen hierzu gibt es im deutschsprachigen Raum für den Bereich Public Health bisher nicht.

**Methoden:**

Im Rahmen des Projektes *Improving Health Monitoring in Migrant Populations (IMIRA) *am Robert Koch-Institut wurde eine Handreichung zu antidiskriminierender Sprache in der Forschung zu Migration und Gesundheit entwickelt, die aus einem Leitfaden und einer Übersicht über relevante Begriffe und Konzepte besteht. Die Bedarfe, Inhalte und Form dazu wurden in einem Aktionsforschungsprozess mit Projektmitarbeitenden aus dem *IMIRA*-Projekt erarbeitet.

**Ergebnisse:**

Der Leitfaden zeigt 5 Grundprinzipien für einen antidiskriminierenden Sprachgebrauch auf: 1. Generalisierungen und Verallgemeinerungen vermeiden, 2. diskriminierungssensibel formulieren, 3. Selbst- und Fremdbezeichnungen berücksichtigen, 4. Begriffe unterliegen einem ständigen Wandel und 5. eigene Unsicherheiten offen kommunizieren. Die Übersicht, welche online als „Living Document“ zur Verfügung steht, beinhaltet Begriffe und Konzepte, die im Zusammenhang mit dem Thema Gesundheit und Migration häufig verwendet werden.

**Fazit:**

Die Handreichung soll Forschende dafür sensibilisieren und dabei unterstützen, Sprache antidiskriminierend anzuwenden. Dies geht mit einer Reflexion der eigenen Sprache einher und stärkt verantwortungsvolle Forschung zum Thema Migration und Gesundheit. Die Nutzung und der Nutzen der Handreichung können Gegenstand zukünftiger Evaluationen sein.

**Zusatzmaterial online:**

Zusätzliche Informationen sind in der Online-Version dieses Artikels (10.1007/s00103-022-03620-0) enthalten.

## Abstract (en)

**Background:**

Public health research has increasingly focused on migration as a determinant of health. Responsible research in this area requires an anti-discriminatory approach in its conduct, reporting and dissemination. A discrimination-sensitive use of language is a central element. Guidelines in this regard do not yet exist for the field of public health in German-speaking countries.

**Methods:**

Within the framework of the project Improving Health Monitoring in Migrant Populations (IMIRA) at the Robert Koch Institute, a guideline on anti-discriminatory language in research on migration and health was developed. It consists of a manual and an overview of relevant terms and concepts. The needs, content and form of the guideline were developed in an action research process with project staff from the IMIRA project.

**Results:**

The manual shows the following five basic principles for anti-discriminatory language use: (1) avoid generalisations, (2) formulate in a discrimination-sensitive way, (3) use self-designations and external designations, (4) recognise that terms are subject to constant change and (5) openly communicate oneʼs own uncertainties. The overview, which is available online as a “living document”, contains terms and concepts that are frequently used in association with the topic of migration.

**Conclusion:**

The guideline is intended to support researchers in using language in an anti-discriminatory way. This goes hand in hand with a reflection on one’s own language use and strengthens responsible research on the topic of migration and health.

## Hintergrund

Migration als Determinante der Gesundheit ist ein wichtiger Gegenstand der Gesundheitsforschung [1–5]. Rassistische und andere Diskriminierungsformen sollten dabei berücksichtigt werden, denn sie stehen in engem Zusammenhang mit struktureller Benachteiligung und somit auch mit sozioökonomischer Ungleichheit. Als gesundheitliche Ungleichheit bezeichnen wir sozioökonomische und soziale Unterschiede in Gesundheitszustand, Gesundheitsverhalten und in der Gesundheitsversorgung der Bevölkerung [6]. Rassismus und Diskriminierung (Begriffserklärungen siehe Infobox) haben nicht nur einen direkten Effekt auf die Gesundheit, sondern (re)produzieren Barrieren im Zugang zu gesellschaftlicher Teilhabe (z. B. Bildung, Arbeits- und Wohnungsmarkt, Gesundheitsversorgung; [7–11]). Daten des Statistischen Bundesamtes (Destatis), der Organisation für wirtschaftliche Zusammenarbeit und Entwicklung (OECD) und des Sozio-oekonomischen Panels (SOEP) zeigen zum Beispiel, dass Menschen, die der Kategorie „Menschen mit Migrationshintergrund“ gemäß der Definition des Statistischen Bundesamtes zugeordnet werden, in Deutschland überproportional häufig von Armut und Arbeitslosigkeit betroffen sind und seltener über einen Berufsschul- oder Hochschulabschluss verfügen als Menschen, die dieser Kategorie nicht zugeordnet sind [12–14]. Da soziale Benachteiligung und Krankheitsrisiken miteinander assoziiert sind [4], stellen Diskriminierung und Rassismus relevante Determinanten der Gesundheit dar. In der internationalen Forschung wird der Einfluss direkter, institutioneller und struktureller Formen von (rassistischer) Diskriminierung auf die Gesundheit bereits intensiv beforscht [15–17].

In der COVID-19-Pandemie kamen gesundheitliche Ungleichheiten verstärkt zum Vorschein. Menschen aus sozioökonomisch benachteiligten Bevölkerungsgruppen haben aufgrund prekärer Lebens‑, Arbeits- und Wohnbedingungen bei teilweise fehlender sozialer Absicherung [18] ein erhöhtes SARS-CoV-2- bzw. COVID-19-assoziiertes Infektions‑, Hospitalisierungs- und Sterberisiko [10, 19]. Dabei zeigte sich, dass migrationsbedingte Faktoren das Infektionsgeschehen vor allem in frühen Phasen der Pandemie beeinflussten, während in späteren Phasen sozioökonomische Ungleichheiten eine größere Rolle spielten [20]. In der öffentlich-medialen Berichterstattung standen jedoch oftmals nicht die strukturellen Risikofaktoren im Fokus, sondern die vermeintliche Herkunft der Betroffenen, deren (vermeintliche) Zugehörigkeit zu bestimmten ethnischen Gruppen sowie zugeschriebene kulturelle Praktiken und Verhaltensmuster. Damit werden bewusst oder unbewusst kulturalisierende Muster [5, 21] genutzt und relevante soziale Determinanten im Diskurs verschleiert. Die Reproduktion rassistischer Zuschreibungen sowie die Abwertung und Diskriminierung von Bevölkerungsgruppen können dadurch befördert werden [22].

### Warum ist antidiskriminierende Sprache in der Gesundheitsforschung wichtig?

Sprache ist in der Forschung zentral für das Verfassen von Publikationen, Projektberichten, Präsentationen, aber auch für die Kommunikation im Team sowie mit Forschungspartner*innen und Studienteilnehmenden. Mit der verwendeten Sprache werden jedoch auch Bilder und Inhalte übertragen [23], die jenseits einer scheinbaren Neutralität wirkmächtige Botschaften, implizite normative Bewertungen oder Ideologien beinhalten oder reproduzieren können [24]. Dazu gehören auch Begriffe und Kategorisierungen, die ausgrenzende oder abwertende Botschaften beinhalten können. Aber wie können Individuen und Gruppen antidiskriminierend beschrieben und benannt werden? Wie kann sichergestellt werden, dass Sprache keine Stereotypisierungen (re)produziert?

Diese Fragen stellten wir uns auch im Rahmen des Projektes *Improving Health Monitoring in Migrant Populations *(*IMIRA*). Eines der Ziele von *IMIRA* war es, eine verbesserte Einbindung von der bisher im Rahmen von Surveys unterrepräsentierten heterogenen Gruppe von Menschen mit Migrationsgeschichte in das Gesundheitsmonitoring am Robert Koch-Institut (RKI) zu erreichen [25, 26], um in zukünftigen Studien die Diversität der Bevölkerung besser abzubilden und so repräsentative Aussagen über die Gesundheit der gesamten in Deutschland lebenden Bevölkerung zu treffen. Eine verantwortungsvolle Sprache in der Kommunikation mit Adressat*innen z. B. in Einladungsschreiben, Befragungen sowie bei der Dissemination von Forschungsergebnissen stellte sich im Projekt als wichtiges Element und Ziel heraus, welches zugleich Herausforderungen und Unsicherheiten mit sich brachte. So kann zum Beispiel die in bisherigen Forschungsprojekten benutzte Kategorie „Menschen mit Migrationshintergrund“ exkludierende Botschaften übertragen [27].

Im Forschungsteam wurden diese Fragen reflektiert und entschieden, erstmalig im deutschsprachigen Raum eine Handreichung für einen diskriminierungssensiblen Sprachgebrauch in der Public-Health-Forschung zu entwickeln. Mit der Vorstellung der Handreichung im vorliegenden Artikel soll zum einen eine Hilfestellung für Public-Health-Praxis und -Forschung angeboten werden. Gleichzeitig soll sie zu einer Diskussion beitragen, die Antidiskriminierung und diskriminierungssensible Sprache als Bestandteil verantwortungsvoller Forschung versteht und voranbringt.

## Methoden

Als Forschungsmethode zur Identifikation von Unsicherheiten bei Forschenden im Sprachgebrauch in Bezug auf Migration und Gesundheit und vorhandener Unterstützungsbedarfe wurde der Ansatz der Aktionsforschung gewählt [28]. Hier beteiligen sich Akteur*innen, die ihre eigene Praxis als Forschungsfeld erforschen. Indem Daten gemeinsam erhoben und analysiert werden, können unmittelbar aus der Praxis heraus Handlungsbedarfe identifiziert und Veränderungen bereits während des Forschungsprozesses angestoßen werden [29, 30].

Das vorliegende Forschungsvorhaben wurde von einer wissenschaftlichen Mitarbeiterin aus dem *IMIRA*-Projekt angestoßen, angeleitet und ausgewertet. Die von ihr definierten Forschungsfragen wurden im Sinne der Aktionsforschung im Juni und Juli 2017 mit 5 Kolleg*innen aus dem Projektteam in 2 von ihr moderierten Fokusgruppendiskussionen à 90 min kritisch diskutiert. Der Einsatz von Fokusgruppen ermöglicht die gemeinsame explorative Erfassung von Perspektiven und Sichtweisen der Teilnehmenden [31]. Diese können entsprechend den eigenen Biografien, Bildung und Erfahrungen unterschiedlich sein. Im Rahmen der Diskussion kann flexibel auf neue, in der Gruppe entstehende Themenfelder und Diskussionsinhalte eingegangen werden [32]. Die in diesem Kontext erhobenen Daten dienten als Grundlage für die Entscheidung bezüglich einer geeigneten Form der Handreichung und deren Inhalt.

Durch dieses Vorgehen wurden Projektmitarbeiter*innen zu an der Aktionsforschung partizipierenden Personen beziehungsweise zu Forschenden im Prozess. Sie wurden im Vorfeld telefonisch und am Tag der Fokusgruppe „face to face“ über das Vorgehen und die Ziele der Forschung informiert und haben sich im Sinne eines Safe Space gegenseitig zur Verschwiegenheit über die besprochenen Aspekte verpflichtet. Aussagen aus den Fokusgruppen sind nicht auf die Personen zurückführbar, da weder personenbezogene Daten erfasst noch Aussagen in den Notizen mit einzelnen Personen verknüpft wurden.

Die Datenauswertung erfolgte auf der Grundlage schriftlicher Protokolle (Transkripte) sowie weiterer in den Fokusgruppen generierter Daten (Flipcharts, erstellte Notizen durch die Teilnehmenden) in Anlehnung an die Auswertungsmethode der qualitativen Inhaltsanalyse nach Kuckartz [33]. Nach der Sichtung des Datenmaterials wurde dieses in einem induktiven Vorgehen kodiert und in Kategorien zusammengefasst.

Zeitgleich erfolgte eine Bestandsaufnahme der Literatur zu antidiskriminierender Sprache und bereits bestehenden Orientierungsmaterialien. Dazu wurde eine Literaturrecherche in der Datenbank PubMed (Suchstrategie unter Verwendung der kombinierten MeSH-Terms „terminology“, „discrimination“, „glossary“, „migration“, „public health“) und in der Suchmaschine Google Scholar vorgenommen. Die Ergebnisse wurden durch Beratung von Expert*innen im Forschungsfeld Migration und Gesundheit ergänzt.

Basierend auf den Erkenntnissen aus der Literatur und den Ergebnissen der Fokusgruppen wurde der *Leitfaden zu diskriminierungssensibler Sprache *erstellt. Der iterative kontinuierliche Einbezug weiterer Kolleg*innen verfolgte das Ziel, die Inhalte des Leitfadens adressat*innengerecht zu gestalten.

Zur Erstellung der dazugehörigen *Übersicht über relevante Begriffe und Konzepte* wurde eine Sichtung wissenschaftlicher Publikationen im Bereich Migration und Gesundheit durchgeführt, um in dem Kontext häufig genutzte Begriffe zu identifizieren. Anschließend wurde auf Grundlage bestehender Glossare [34–37] ein an die Bedarfe der Gesundheitsforschung (im deutschsprachigen Raum) angepasstes Dokument erstellt. Als Kriterium diente der Leitfaden mit seinen 5 Grundprinzipien.

Der Prozess der Erstellung der Handreichung wurde durch externe Expert*innen aus der rassismuskritischen Forschung und Praxis inhaltlich beraten und unterstützt.

## Ergebnisse

### Bestandsaufnahme der Literatur

Im Bildungs- und Medienbereich wird die Nutzung antidiskriminierender Sprache bereits seit Langem durch Orientierungshilfen und Glossare unterstützt [34–36]. Für die Public-Health-Forschung hingegen wurden nur 3 Publikationen aus dem englischsprachigen Raum gefunden.

In *Glossary of terms relating to ethnicity and race: for reflection and debate* (2004) beschreibt Raj Bhopal aus der Perspektive einer Person of Colour am Beispiel verschiedener englischer Begriffe (z. B. *Asian*) Limitationen und Probleme von Gruppenbezeichnungen bestimmter in der Regel nicht *weißer* ethnischer Gruppen in der Forschung und weist auf die Notwendigkeit der Nutzung international abgestimmter Konzepte und Begriffe hin. Auch betont er, dass publizierte Forschungsergebnisse immer einen Nutzen für beforschte Bevölkerungsgruppen haben müssen und Reproduktion von Rassismus oder Stigmatisierung vermieden werden muss [38]. Zu dem Schluss, dass es bisher keinen international abgestimmten Konsens zu rassismus- und migrationsbezogenen Konzepten in der epidemiologischen Forschung gibt und die Terminologie je nach historischem, geografischem oder theoretischem Kontext sehr unterschiedlich ist, kommen auch Urquia und Gagnon in der Publikation *Glossary: migration and health* von 2011 [39]. Auch sie machen dies anhand von Beispielen deutlich und fordern Forschende auf, von der Nutzung bestimmter Kategorien abzusehen, wie beispielsweise „illegal migrant“ oder „acculturation“, und deren Aussage- und Informationswert kritisch zu hinterfragen.

2019 reagierten Johnson und Kolleg*innen mit *A glossary for the first World Congress on Migration, Ethnicity, Race and Health* darauf, dass, wie bereits von Bhopal, Urquia und Gagnon beschrieben, ein, wie sie es nennen, allgemein anerkanntes Glossar im Kontext der Forschung zu Migration und Gesundheit fehlt und in Texten teilweise Begriffe und Konzepte genutzt werden, die als unangemessen oder abwertend betrachtet werden [40]. Sie bieten mit dem Glossar eine Hilfestellung an, um ein gemeinsames Verständnis zu fördern und eine konstruktive Diskussion anzuregen. Das Glossar geht über die Begriffe und Konzepte der beiden anderen Glossare hinaus und greift u. a. Konzepte wie „discrimination“, „equity“ und „racism“ auf.

### Fokusgruppendiskussionen

Die erste Fokusgruppe ermöglichte einen Einstieg in das Themenfeld und hatte das Ziel, Erwartungen und Bedarfe im Hinblick auf eine sprachliche Orientierungshilfe zu identifizieren. Die zweite Fokusgruppe diente der weiteren inhaltlichen Ausarbeitung und war entlang folgender Leitfragen angelegt: 1) *Welche Unsicherheiten und Herausforderungen habe ich selbst bei der Anwendung von Sprache erlebt?* und 2) *Welche Empfindungen hat Sprache in mir ausgelöst?*

#### Leitfaden zu diskriminierungssensibler Sprache im Bereich Migration und Gesundheit.

In den Fokusgruppen wurde der Bedarf für ein Dokument identifiziert, welches 1) zur Selbstreflexion und Sensibilisierung des eigenen Handelns in der Public-Health-Forschung einlädt und motiviert, 2) keine Handlungsanweisung, sondern eine Orientierung für den Sprachgebrauch bietet und 3) niedrigschwellig zugänglich und möglichst leicht in der Praxis anwendbar ist. Hierfür wurden aus der Literatursichtung und den Fokusgruppen die folgenden 5 Grundprinzipien für einen antidiskriminierenden Sprachgebrauch identifiziert:

1. Generalisierungen und Verallgemeinerungen vermeiden,
2. diskriminierungssensibel formulieren,
3. Selbst- und Fremdbezeichnungen berücksichtigen,
4. Begriffe unterliegen einem ständigen Wandel und
5. eigene Unsicherheiten offen kommunizieren.

Die Grundprinzipien wurden in einem „Leitfaden zu diskriminierungssensibler Sprache im Bereich Migration und Gesundheit“ beschrieben (Stand 20.10.2022, Abb. 1). Das Dokument ist auf der RKI-Website^1^ zu finden.

**Figure 1:**
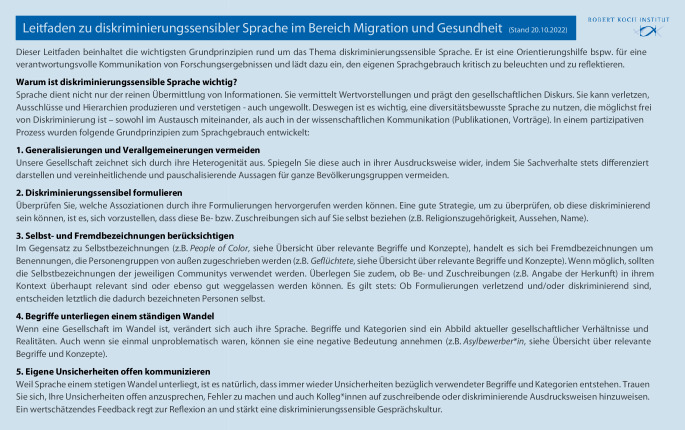

#### Übersicht über relevante Begriffe und Konzepte zum diskriminierungssensiblen Sprachgebrauch.

Neben dem Leitfaden wurde die Notwendigkeit einer ergänzenden Übersicht über relevante Begriffe und Konzepte als praktische Handlungshilfe identifiziert. Es wurde daraufhin die „Übersicht über relevante Begriffe und Konzepte zum diskriminierungssensiblen Sprachgebrauch rund um das Thema Migration und Gesundheit“ erstellt. Da Begriffe in ihren Bedeutungen und ihrer Verwendung einem ständigen Wandel unterliegen, wurde beschlossen die Übersicht online als „Living Document“ (lebendiges Dokument) zur Verfügung zu stellen, um Lesenden die Möglichkeit zu geben, die Formulierungen mitzugestalten und es regelmäßig aktualisieren zu können.

Die Übersicht (Stand 20.10.2022, siehe Onlinematerial zu diesem Beitrag) enthält aktuell 33 Begriffe und 17 Konzepte, die in der Forschung zu Migration und Gesundheit häufig verwendet werden, sowie Hinweise zu problematischen Formulierungen, die aus der Perspektive einer diskriminierungssensiblen Kommunikation nicht verwendet werden sollten. Die differenzierte Benennung von Gruppen bzw. Minderheiten und Mehrheiten findet ebenso Beachtung wie die Selbstbezeichnungen von Gruppen und migrationsassoziierte Konzepte der Gesundheitswissenschaften. Für Begriffe, die aufgrund des Risikos, Stereotype oder Rassismus zu reproduzieren, nicht (mehr) verwendet werden sollten, werden Alternativen vorgeschlagen. Da es in anderen Disziplinen gut entwickelte deutschsprachige Glossare gibt, wurde sich in der Definition der ausgewählten Begriffe auf diese bezogen.

## Diskussion

Das Anfertigen von Texten und Publikationen erfordert die stetige Überprüfung und Aktualisierung der verwendeten Sprache sowie eine Auseinandersetzung mit den eigenen Erfahrungen, Prägungen und Positionierungen [35, 41]. Eine Reflexion verwendeter Begriffe und Konzepte kann Forschende dabei unterstützen, verantwortungsvoll und diskriminierungssensibel zu kommunizieren [42].

Eine verantwortungsvolle Forschung im Bereich Migration und Gesundheit verfolgt das Prinzip der Nichtschädigung und beinhaltet folglich auch den Gebrauch einer diskriminierungssensiblen Sprache [38, 43]. Die hier vorgestellte Handreichung besteht aus einem Leitfaden und einer Übersicht mit Begriffen und Konzepten, die im Bereich Migration und Gesundheit relevant sein können. Sie sollen einen diskriminierungssensiblen Sprachgebrauch in der Forschung unterstützen und laden dazu ein, den eigenen Sprachgebrauch zu reflektieren, eigene Unsicherheiten offen zu benennen und nachzufragen. Die 5 im Leitfaden vorgestellten Grundprinzipien zeigen Fallstricke der Sprache auf, betonen, dass Begriffe und Konzepte je nach Perspektive unterschiedliche Bedeutung und Konsequenzen für einzelne Menschen oder Gruppen haben können, und weisen darauf hin, dass sich Sprache dynamisch weiterentwickelt.

### Wissenschaft braucht Kategorien.

Auch wenn der Leitfaden eine differenzierte Darstellung und Kommunikation, die Vermeidung von Generalisierungen und die Bevorzugung von Selbstbezeichnungen empfiehlt, ist er nicht als Plädoyer für die gänzliche Abschaffung von Kategorien und Konzepten in der Forschung zu verstehen, sondern für ihre fortwährende Reflexion.

Wissenschaftlich oder administrativ etablierte Konzepte, wie der „Migrationshintergrund“ im deutschsprachigen oder „race“ und „ethnicity“ im englischsprachigen Raum, sind Instrumente in der Public-Health-Forschung. Durch ihre Nutzung sollen Unterschiede auf Gruppenebene in Bezug auf den Gesundheitszustand und die Inanspruchnahme der Gesundheitsversorgung aufgezeigt werden. Präventionsbedarfe z. B. im Hinblick auf einen verbesserten Schutz vor einer SARS-CoV-2-Infektion von Bevölkerungsgruppen verschiedener ethnischer Zugehörigkeiten konnten auf Grundlage solcher Konzepte im Vereinigten Königreich zeitnah identifiziert werden [44].

Gleichzeitig müssen sich Forschende darüber bewusst sein, dass die Nutzung solcher Konzepte diskriminierende und rassistische Bilder und Vorurteile (re)produzieren kann [22], insbesondere wenn sie in einem sozialen, politischen und öffentlichen Umfeld erfolgt, in dem eine migrationsfeindliche (Grund‑)Einstellung (vor‑)herrscht. Die ethische Rechtfertigung für die Nutzung von Konzepten wie „Migrationshintergrund“, „race“ und „ethnicity“ muss, so Bhopal, verantwortungsvoll erfolgen und die Verbesserung der Gesundheit aller Bevölkerungsgruppen zum Ziel haben [43].

### Konzepte kritisch betrachtet.

Die vorliegende Handreichung hat neben ihrer Funktion als praktische Orientierungshilfe in der Public-Health-Forschung zu Migration und Gesundheit ein weiteres Ziel: Sie soll dazu beitragen, dass die in der Forschung zu Migration und Gesundheit angewendeten Begriffe und Konzepte im Hinblick auf eine verantwortungsvolle Kommunikation immer wieder kritisch reflektiert und angepasst werden.

Ein Beispiel hierfür ist das in Deutschland seit 2005 und auch in den repräsentativen bevölkerungsbezogenen Gesundheitssurveys genutzte – wenn auch immer mal wieder anders operationalisierte – Konzept „Migrationshintergrund“. Es steht in der Kritik, keine Selbstbezeichnung, sondern eine Fremdzuschreibung zu sein, die zu einem „Othering“ beiträgt, einer Kennzeichnung als „Andere“, „Fremde“, „Nichtdazugehörige“ von deutschen und nichtdeutschen Menschen mit Migrationsbiografie [27, 45, 46]. Zudem ist es mit keiner in anderen Ländern genutzten Kategorie vergleichbar und hat keine Aussagekraft hinsichtlich Diskriminierungs- und Rassismuserfahrungen oder struktureller Ausschlüsse und daraus resultierender sozioökonomischer und gesundheitlicher Ungleichheiten [45, 46]. Die Fachkommission Integrationsfähigkeit hat daher 2020 vorgeschlagen, in Zukunft die Kategorie „Eingewanderte und ihre (direkten) Nachfahren“ zu nutzen [47], wobei auch diese wegen ihres Abstammungsfokus kritisch diskutiert wird [27].

### Der Nutzen diskriminierungssensibler Sprache für die Forschung.

Bisher hat die Bedeutung diskriminierungssensibler Sprache in der Gesundheitsforschung wenig Aufmerksamkeit bekommen, auch wenn es einzelne Anstöße für die Notwendigkeit international abgestimmter Glossare gab [38–40, 48]. Das *Glossary on Migration Law *der *International Organization of Migration* (IOM) beispielsweise stellt eine umfangreiche Sammlung migrationsbezogener Terminologien mit einem juristischen Schwerpunkt zur Verfügung. Gleichzeitig beinhaltet es eine kritische Reflexion von Begriffen (wie z. B. „illegal migrant“) und weist darauf hin, dass im öffentlichen Diskurs verwendete Begriffe maßgeblich die Art und Weise prägen, wie die Gesellschaft Migration wahrnimmt [48].

In Ergänzung dazu wird auch eine Auseinandersetzung benötigt, die sich dem spezifischen deutschsprachigen Kontext widmet. Während im deutschsprachigen Raum die gendersensible Sprache in Forschungsanträgen, wissenschaftlicher Kommunikation und Publikationen inzwischen als Ergebnis intensiver und langjähriger Auseinandersetzung mit diesem Thema weitestgehend zum Standard gehört, werden andere Diskriminierungsdimensionen, wie z. B. ethnische oder soziale Herkunft, Behinderung oder sexuelle Identität, in der wissenschaftlichen Sprache selten berücksichtigt oder thematisiert.

Eine diversitätsorientierte und diskriminierungssensible Sprachanwendung kann z. B. bei der Adressierung von unterschiedlichen Gesundheitsbedarfen in der Bevölkerung hilfreich sein. Sie kann bei der Studienplanung, neben der Nutzung von mehrsprachigen Informationsmaterialien, dabei helfen, dass Menschen besser erreicht werden, sich angesprochen und nicht stigmatisiert fühlen [25]. Auch die Einplanung von personellen und finanziellen Ressourcen zur Einbindung von Expertisen aus beforschten Bevölkerungsgruppen kann hilfreich sein, um diskriminierungssensibel vorzugehen. Beispielsweise kann dies durch ein Community-Board geschehen, welches während des gesamten Prozesses die Projektplanung und Durchführung auch im Hinblick auf die Nutzung von Begriffen und Konzepten begleitet und berät.

### Schutz und Beteiligung beforschter Bevölkerungsgruppen.

Ethische Grundprinzipien in der Forschung sollen sicherstellen, dass beforschte Personen und Bevölkerungsgruppen geschützt sind. Ihnen darf durch Forschung kein Schaden widerfahren [49–51]. Daher ist Antidiskriminierung nicht nur in der Sprache, sondern in der Forschung generell eine wichtige Voraussetzung für verantwortungsvolles Handeln. Zentral sind dafür die Berücksichtigung und Benennung von gesellschaftlichen Verhältnissen und Strukturen und der damit verbundenen sozialen Determinanten der Gesundheit, wie z. B. berufliche Situation, aufenthaltsrechtliche Bestimmungen oder Zugang zur Gesundheitsversorgung. Auch auf der Praxisebene ist Antidiskriminierung bei der Planung und Umsetzung von Public-Health-Maßnahmen mitzudenken [52]. Dies erfordert eine reflektierte Haltung in Forschung und Praxis. Die Beteiligung von beforschten bzw. betroffenen Individuen und Gruppen ist eine weitere Möglichkeit, um wichtige Aspekte der Verantwortung und Ethik zu stärken und zu gewährleisten. Durch Beteiligung können nicht nur Zugänge geschaffen werden, sondern auch u. a. ein respektvolles Vorgehen gegenüber Einzelpersonen und Gemeinschaften gestärkt, ein angemessener Nutzen abgeschätzt und Schaden minimiert werden [53]. In den USA, im Vereinigten Königreich und Kanada sowie in vielen Regionen des globalen Südens wie Südafrika, Indien, Brasilien und China ist die Beteiligung beforschter und betroffener Gruppen an Forschung und Praxis schon längst etabliert [54].

### Antidiskriminierung voranbringen.

Auch eine antidiskriminierende Sprache innerhalb Institutionen sowie Antidiskriminierung generell in institutionellen Strukturen unterstützen verantwortungsvolle Forschung. Antidiskriminierung und Diversität sind Themen, die mittlerweile in der Organisationsentwicklung einen hohen Stellenwert haben. Wenn die Diversität der Gesellschaft sich auch in Forschungsinstituten widerspiegelt, kann verantwortungsvolle Public-Health-Forschung durch unterschiedliche Perspektiven, Biografien und Erfahrungen gestärkt werden. Ferner können Antidiskriminierungstrainings für Mitarbeitende die Thematisierung von Antidiskriminierung im wissenschaftlichen Diskurs sowie eine verantwortungsvolle Dissemination von Forschungsergebnissen unterstützen.

### Limitationen

Eine zentrale Limitation des Vorhabens ergibt sich daraus, dass die an der Aktionsforschung beteiligten Personen sich bereits mit den Themen Antidiskriminierung und Rassismus befasst hatten und das Thema der antidiskriminierenden Sprache für wichtig erachteten. Perspektiven aus der Praxis oder anderen Forschungseinrichtungen konnten hier nicht abgebildet werden. Ebenso fehlt die Perspektive von Personen, die sich nicht mit dem Diskurs um Antidiskriminierung befassen. Die Berücksichtigung weiterer Perspektiven wäre jedoch wichtig, um identifizieren zu können, welche weiteren Inhalte und Formen es braucht, um das Thema breit implementieren zu können.

## Fazit

Insgesamt konnte sich mit der hier vorgestellten Handreichung im Rahmen des *IMIRA*-Projekts, dem bislang größten Projekt am RKI zum Thema Migration, den Themen Verantwortung und Sprache in der Forschung angenommen und ein Impuls zur weiteren Stärkung des Themas in der Forschung gesetzt werden. Unsicherheiten im Sprachgebrauch und der daraus resultierende Bedarf für eine Handreichung wurden erst im Verlauf des *IMIRA*-Projekts identifiziert bzw. dargelegt. Auch wenn die Entwicklung einer Handreichung zu antidiskriminierender Sprache im Kontext Migration in der initialen Projektplanung nicht vorgesehen war, konnte das Vorhaben dennoch unter Beteiligung von Co-Forschenden und unter Einbezug externer Expert*innen umgesetzt und die gewonnenen Erkenntnisse bereits im Projektverlauf genutzt werden.

Eine Möglichkeit für die Integration antidiskriminierender Maßnahmen in der Gesundheitsforschung ist die Abkehr von der Erfassung des Migrationshintergrundes hin zur Erfassung von migrationsspezifischen Variablen wie des Aufenthaltsstatus oder der Sprachkenntnisse sowie der Entwicklung eines Instruments zur Erfassung subjektiv wahrgenommener Diskriminierung, welches in Zukunft im bundesweiten Gesundheitsmonitoring eingesetzt werden soll [25, 26]. Ein weiterer wichtiger Schritt in diese Richtung wurde in Deutschland mit dem *Afrozensus *gemacht, der ersten durch Schwarze, afrikanische und afrodiasporische Menschen selbst organisierten bundesweiten Onlinebefragung zu Lebensrealitäten, Diskriminierungserfahrungen und Perspektiven [55].

Die Beschäftigung mit dem Thema antidiskriminierende Sprache in der Public-Health-Forschung zu Migration macht deutlich, dass verantwortungsvolle Sprache Forschung unterstützt, Zugänge schafft und beforschte Menschen und Bevölkerungsgruppen schützen kann. Die hier vorgestellte Handreichung zum Sprachgebrauch im Forschungsbereich Migration und Gesundheit ist eine Orientierungshilfe und kann als Impuls für eine Diskussion verstanden werden, um Antidiskriminierung in der Public-Health-Forschung in Zukunft noch stärker zu verankern. Die Nutzung und der Nutzen der Handreichung können Gegenstand zukünftiger Evaluationen sein. Aus einer intersektionalen Perspektive heraus sollten weitere Diversitätsdimensionen, wie beispielsweise soziale Herkunft oder Behinderung für eine diskriminierungssensible Sprache im Public-Health-Bereich, Berücksichtigung finden.

### Infobox Begriffserklärungen: Rassismus und Diskriminierung

**Rassismus**

„Wenn strukturell benachteiligte Gruppen oder einzelne Menschen aufgrund tatsächlicher oder vermeintlicher körperlicher oder kultureller Merkmale (z. B. Hautfarbe, Herkunft, Sprache, Religion) pauschal abgewertet und ausgegrenzt werden“, spricht man von Rassismus (Definition nach dem Glossar der Neuen Deutschen Medienmacher*innen https://glossar.neuemedienmacher.de/glossar/).

**Diskriminierung**

„Diskriminierung ist die ungleiche, benachteiligende und ausgrenzende Behandlung von Gruppen und Individuen ohne sachlich gerechtfertigten Grund. Diskriminierung kann sich zeigen als Kontaktvermeidung, Benachteiligung beim Zugang zu Gütern und Positionen, als Boykottierung oder als persönliche Herabsetzung. Der Begriff bezeichnet sowohl den Vorgang als auch das Ergebnis. Die Durchsetzung von Diskriminierung setzt in der Regel soziale, wirtschaftliche, politische oder publizistische Macht voraus“ (Definition nach dem Glossar des Informations- und Dokumentationszentrums für Antirassismusarbeit e. V. – IDA).

## Supplementary Information





## Footnotes

1 https://www.rki.de/DE/Content/GesundAZ/M/Migration_Gesundheit/Handreichung.html; Zugriff: 23.10.2022.
