# Supplementary material for: A guideline on discrimination-sensitive language in research on migration and health
Source: Bundesgesundheitsblatt Gesundheitsforschung Gesundheitsschutz. 2022 Nov 21;65(12):1316–23. [Article in German] doi: 10.1007/s00103-022-03620-0 (PMC9684915; doi:10.1007/s00103-022-03620-0)
Supplement: Supplementary file 1 [file 103_2022_3620_MOESM1_ESM.pdf]

Übersicht über relevante Begriffe und Konzepte zum diskriminierungssensiblen Sprachgebrauch rund um das Thema Migration und Gesundheit (Stand: 20.10.2022)

Inhaltsverzeichnis

Übersicht über relevante Begriffe und Konzepte zum diskriminierungssensiblen Sprachgebrauch rund um das Thema Migration und Gesundheit 1

Informationen zum vorliegenden Dokument 3

Begriffserklärung 3

Afrodeutsch 3

Asylbewerber*in 4

Asylant*in 4

Ausländer*in 5

Ausländer*in mit deutschem Pass 5

Aussiedler*in 5

Binnengeflüchtete 6

Displaced Person 6

Einheimische 6

Einwanderungsgesellschaft 6

Ethnie/Ethnische Gruppe 7

Ethnizität 7

Farbig/Dunkelhäutig 7

Flüchtling 8

Gastarbeiter*in 8

Geflüchtete 9

Menschen mit Migrationshintergrund 9

Menschen ohne Migrationshintergrund 9

Migrant*in 10

Moslem, Moslems 10

Muslim, Muslima, Muslime 10

Person/People of Color (PoC) 10

Rom*nja, Roma 11

Schutzsuchende 11

Schwarze Deutsche 12

Schwer erreichbare Gruppen (hard-to-reach) 12

Sinti*izze 13

Spätaussiedler*in 13

Menschen mit Sprachproblemen (auch: keine oder schlechte Sprachkenntnisse, fehlende Sprachkenntnisse, schlechte Sprachfähigkeiten) ... 13
Türkischstämmig ... 14
weiß ... 14
Xenophobie ... 14
Zuwanderungsgesellschaft oder Aufnahmegesellschaft/ Aufnahmeland ... 14
Konzepte ... **15**
Akkulturation ... 15
Antidiskriminierung ... 15
Antiziganismus ... 15
Antiromaismus ... 16
Diskriminierung ... 16
Diversität ... 16
Integration ... 17
Intersektionalität ... 17
Kolonialismus ... 17
Kultur ... 17
Kulturalisierung ... 18
Marginalisierte Bevölkerungsgruppen ... 18
Othering ... 19
Parallelgesellschaft ... 19
Partizipation ... 19
Rasse/Race ... 19
Rassismus ... 20
Hinweise zu problematischen Formulierungen ... 20
Begriffe mit Endungen wie „-strom/ströme“, „-schwemme“, „-influx“ oder „-welle“ ... 20
Begriffe mit der Endung „-krise“ ... 21
Kennzeichnung von Menschen als „illegal“ ... 21
Literatur ... 21

Informationen zum vorliegenden Dokument

Im Rahmen des Projekts „Improving Health Monitoring in Migrant Populations“ (IMIRA) wurde am Robert Koch-Institut in Zusammenarbeit mit externen Akteuren mit entsprechender inhaltlicher Expertise (Facheinrichtungen, Forschung, Selbstorganisation) und unter Berücksichtigung des bestehenden Diskussionsstandes eine Handreichung zu antidiskriminierender Sprache in der Forschung zu Migration und Gesundheit entwickelt. Diese besteht aus einem Leitfaden und der hier vorliegenden Übersicht über relevante Begriffe und Konzepte, die in Veröffentlichungen in diesem Themenfeld Anwendung finden. Begriffe, die Rassismus und Ausschlüsse reproduzieren, sind entsprechend beschrieben. Sofern vorhanden, sind alternative Formulierungsvorschläge genannt. Bei der Erstellung wurde sich an bereits bestehenden Glossaren aus andere Fachdisziplinen, Organisationen und Institutionen orientiert: Deutsche Aidshilfe, Amnesty International, Neue Deutsche Medienmacher*innen, AntiDiskriminierungsBüro Köln, Amadeu Antonio Stiftung und Informations- und Dokumentationszentrum für Antirassismusarbeit e. V. (IDA). Es wird empfohlen, auch die genannten Glossare bei Bedarf zu nutzen.

Diese Übersicht und Reflexion der Begriffe ist als ein lebendiges Dokument zu verstehen, das fortgehend weiterentwickelt und aktualisiert wird.

Engere juristische Begriffe, z.B. zum Aufenthaltsstatus und Migrationsrecht, können in bereits existierenden Glossaren z.B. des Europäischen Migrationsnetzwerks nachgeschlagen werden, diese Konzepte werden hier nicht erfasst.

Bei diesem Dokument handelt es sich um ein **"Living Document"**. Die aktuellste Verison kann unter dem folgenden Link abgerufen werden: **https://www.rki.de/DE/Content/GesundAZ/M/Migration_Gesundheit/Handreichung.html**

Rückmeldungen gerne an migration@rki.de.

↑ nach oben

Begriffserklärung

Afrodeutsch

Der Begriff wird von Schwarzen Menschen in Deutschland als Selbstbezeichnung verwendet. Ein familiärer Bezug zu Afrika kann, muss aber nicht zwingend bestehen - die familiären Hintergründe können beispielsweise ebenso in den USA oder Deutschland liegen [1].

↑ nach oben

Asylbewerber*in

Juristisch bezeichnet der Begriff eine Person, die beim Bundesamt für Migration und Flüchtlinge einen Antrag auf Asyl gestellt hat, der noch nicht entschieden ist. Als solcher wird er auch als statistischer Begriff genutzt (z. B. Asylbewerberleistungsstatistik). Das Asylrecht für politisch Verfolgte ist in Deutschland in Art. 16 a im Grundgesetz verankert.

Umgangssprachlich werden hiermit aber auch Menschen bezeichnet, auf die diese Situation nicht zutrifft, z. B. weil sie bereits einen Aufenthaltstitel erhalten haben oder noch nicht registriert sind. Zudem wird der Begriff zunehmend im menschenfeindlichen/ rechtspopulistischen Kontext als Beschimpfung genutzt [1, 2].

Vorschlag: Vor der Wahl eines Begriffs müssen wir uns fragen, wen wir genau meinen und ggf. welcher juristisch korrekte Begriff im Einzelfall zutrifft. Entsprechend können z. B. folgende Begriffe in Frage kommen: Asylsuchende, Schutzsuchende (Sammelbezeichnung für eine Reihe an Aufenthaltstiteln), Antragstellende, Geflüchtete, geflüchtete Menschen, Menschen im laufenden Asylverfahren, Schutzberechtigt/Bleibeberechtigt.

↑ nach oben

Asylant*in

Abwertender und negativ konnotierter Begriff für Geflüchtete, die als Bedrohung und Belastung und nicht als Schutzsuchende betrachtet werden [1].

Vorschlag: Asylsuchende, Antragstellende, Menschen im laufenden Asylverfahren, Schutzberechtigte

Hinweis zu Begrifflichkeiten (Bundesamt für Migration und Flüchtlinge)

1. Asylsuchende
    - Personen, die einen Asylantrag stellen wollen, aber noch nicht als Asylantragstellende beim Bundesamt erfasst sind
2. Asylantragstellende
    - Personen, die sich im laufenden Asylverfahren befinden, über deren Verfahren aber noch nicht entschieden ist
3. Schutzberechtigte
    - Personen, die eine Asylberechtigung, einen Flüchtlingsschutz oder subsidiären Schutz erhalten haben

↑ nach oben

Ausländer*in

Juristisch: Eine Person, die keine deutsche Staatsangehörigkeit besitzt. Der Begriff ist kein Synonym für Migrant*innen oder Menschen mit Migrationshintergrund, da viele keine Ausländer*innen sind, sondern deutsche Staatsangehörige [1, 2].

↑ nach oben

Ausländer*in mit deutschem Pass

Sachlich nicht korrekt und in sich widersprüchlich, da die korrekte Verwendung des Begriffes „Ausländer*in“ eine Person ohne deutsche Staatsangehörigkeit beschreibt [3].

Vorschlag: Vermutlich sind hier deutsche Staatangehörige mit Migrationsgeschichte oder Personen mit doppelter Staatsangehörigkeit gemeint

↑ nach oben

Aussiedler*in

Aussiedler*in und Spätaussiedler*in sind deutsche Volkszugehörige, die als deutsche Minderheiten zum Teil seit mehreren Generationen in Ostmitteleuropa, Osteuropa und Zentralasien leb(t)en und nach 1950 nach Deutschland migriert sind. Der Begriff „Aussiedler*in“ wurde bis 1992 verwendet, seit 1993 findet der Begriff „Spätaussiedler*in“ Anwendung. Mit der Erlangung „Statusdeutscheneigenschaft“ erwerben sie die Gleichstellung mit der deutschen Staatsangehörigkeit und gelten nicht als Ausländer*innen [1, 3].

*Alternative: Spätaussiedler*in*

↑ nach oben

Binnengeflüchtete

Von den Vereinten Nationen verwendete Bezeichnung für Personen, die ihren Heimatort verlassen mussten und im selben Land verbleiben (siehe auch Displaced Persons) [1].

↑ nach oben

Displaced Person

Von den Vereinten Nationen verwendete Bezeichnung für Personen, die ihren Heimatort verlassen mussten und im selben Land verbleiben (siehe auch Binnengeflüchtete) [1].

↑ nach oben

Einheimische

Wird häufig als zur Bezeichnung für Menschen ohne Migrationshintergrund verwendet. Dabei wird nicht berücksichtigt, dass auf der einen Seite viele Migrant*innen und People of Color (PoC) und ihre Kinder ebenso in Deutschland einheimisch sind bzw. sich heimisch fühlen und dass als Einheimische bezeichnete Menschen je nach zeitlicher Dimension auch eine Migrationsgeschichte aufweisen können [1].

*Vorschlag: Bürger*innen, Einwohner*innen*

↑ nach oben

Einwanderungsgesellschaft

Gesellschaft, in die Menschen einwandern, um zu bleiben und Teil der Bevölkerung zu werden. Dies ist in Abgrenzung zur Zuwanderungsgesellschaft zu betrachten, die die temporäre Begrenzung der Zugezogenen betont (siehe Zuwanderungsgesellschaft) [18].

↑ nach oben

Ethnie/Ethnische Gruppe

Unter Ethnie wird eine Menschengruppe mit zugeschriebener kollektiver Identität verstanden (Ethnizität). Diese tatsächlichen oder vermeintlichen Gemeinsamkeiten und Verbindungen können sich auf unterschiedliche Aspekte beziehen: z. B. Sprache (wir gehören zusammen, weil wir die gleiche Sprache sprechen), Geschichte (gemeinsame Vergangenheit), Religion (gemeinsamer Glaube), Kultur (geteilte Normen, Werte, Rituale). Von Bedeutung sind auch Vorstellungen von einer gemeinsamen Herkunft („Abstammung“). Dabei ist es nicht entscheidend, ob eine Abstammungsgemeinschaft real vorliegt oder nicht: Die Bezeichnung „Ethnie“ wird vor allem über die Selbstzuschreibung der jeweiligen Gruppe definiert. Als Fremdzuschreibung können ethnisierte Merkmale allerdings auch der Legitimierung von Ausgrenzung und Diskriminierung dienen. Der Begriff wird meistens synonym zu Volk verwendet, auch um letzteren im Sinne eines Euphemismus zu vermeiden. Teilweise ersetzt Ethnie auch den Begriff „Rasse“, ebenfalls um negative Konnotationen dieses Begriffs zu vermeiden [4].

↑ nach oben

Ethnizität

Ethnizität ist die Eigen- oder Fremdzuschreibung von Menschen, einer bestimmten „Ethnie“ anzugehören oder andere „Ethnien“ als separat zu empfinden. Sie ist eine Form kollektiver Identität. Der britische Soziologe Stuart Hall betont in seiner Definition, dass Ethnizität keine feststehende natürliche Eigenschaft ist, sondern als dynamisch und kontextabhängig zu betrachten ist: „Es ist eher eine Überzeugung, eine Vorstellung, eine Bewusstseinsform, die weder natürlich noch ewig ist, sondern zu einem bestimmten Zeitpunkt durch die Umstände hervorgebracht wird. Es ist ein Halt, der dem Individuum das Gefühl gibt, irgendwo in der Welt einen Platz und eine Position zu haben. (…) Ethnizität erinnert uns daran, dass jede/r ‚irgendwo‘ herkommt – ob real oder eingebildet – und dass jede/r das Bedürfnis hat, sich mit etwas zu identifizieren und bei was auch immer zugehörig zu fühlen [4].

↑ nach oben

Farbig/Dunkelhäutig

Eine Fremdbezeichnung, die durch die Kolonialzeit geprägt, rassistisch und negativ konnotiert ist und von vielen als solche bezeichneten Menschen abgelehnt wird [1, 3].

Vorschlag: Schwarze (Deutsche), Afrodeutsche, Black and People of Color (BPoC)

↑ nach oben

Flüchtling

Nach der Genfer Flüchtlingskonvention erhalten Personen Flüchtlingsschutz, deren Leben oder Freiheit in ihrem Herkunftsland aufgrund ihrer Herkunft, Religion, Staatsangehörigkeit, Zugehörigkeit zu einer bestimmten sozialen Gruppe oder aufgrund politischer Überzeugung bedroht sind. Der Begriff umschreibt im engeren, juristischen Sinne nur die Personen, denen nach der Genfer Flüchtlingskonvention Flüchtlingsschutz bereits gewährt wurde, wohingegen er in der Alltagssprache meist aus der Heimat geflohene Personen bezeichnet. Jedoch werden Menschen durch diese Bezeichnung auf einen Teil ihrer Biografie reduziert. Die Endung „–ling" ist zudem oft unmündig konnotiert.

Der Begriff De-facto-Flüchtling ist kein Rechtsbegriff, bezeichnet Personen, die keinen Asylantrag gestellt haben oder eine Ablehnung ihres Asylantrages erfahren haben. Aus humanitären Gründen ist ihnen die Rückkehr in ihr Heimatland nicht zumutbar (Duldung) [1, 2].

Vorschlag: Geflüchtete, geflüchtete Personen/Menschen, geflohene Personen/Menschen, Schutzsuchende, schutzsuchende Personen/Menschen

↑ nach oben

Gastarbeiter*in

Das Wort sollte nur in einem historischen Kontext verwendet werden. Es beschreibt Personen, die ursprünglich für einen zeitlich begrenzten Aufenthalt für eine Erwerbstätigkeit in Deutschland angeworben wurden (1950er- bis 1970er-Jahre). Da viele seitdem dauerhaft in Deutschland leben, ist die Bezeichnung „Gast" unzutreffend [1, 3].

*Vorschlag: Arbeitsmigrant*innen*

↑ nach oben

Geflüchtete

Aktuell ein gut geeigneter Begriff, um Menschen zu benennen, die aus ihrem Land geflohen sind. Da es sich um keinen Begriff der juristischen Fachsprache handelt, kann er auch Menschen einbeziehen, die aus Gründen wie Armut, Krieg, Gewalt oder Klimakatastrophen geflohen sind und keine Anerkennung nach der Genfer Flüchtlingskonvention erhalten [1, 2]. Der Begriff hat sich seit 2015 als Ersatz für den negativ konnotierten und juristisch unzutreffenden Flüchtlingsbegriff etabliert.

Alternative Begriffe: geflüchtete Person, Exilierte, Schutzsuchende

↑ nach oben

Menschen mit Migrationshintergrund

Ein für die amtliche Statistik in Deutschland entwickeltes Konzept. Gemäß der Definition des Statistischen Bundesamtes haben Menschen einen Migrationshintergrund, wenn sie selbst oder mindestens eines ihrer Elternteile nicht mit deutscher Staatsangehörigkeit geboren wurden. Zu dieser Personengruppe zählen zugewanderte und nicht zugewanderte Ausländer*innen, zugewanderte und nicht zugewanderte Eingebürgerte, (Spät-) Aussiedler*innen sowie die als Deutsche geborenen Nachkommen dieser Gruppen. Das Konzept ist eine Fremdzuschreibung und prägt gesellschaftliche Ein- und Ausschlussmechanismen. Als Mensch mit Migrationshintergrund markiert zu werden, kann eine rassistisch- diskriminierende Erfahrung sein. Zudem wird er häufig für die Beschreibung von „Problemgruppen“ verwendet. Es ist zu beachten, dass auch Menschen, die nach der Definition keinen Migrationshintergrund haben, dennoch rassistische Diskriminierung erfahren können, z.B. aufgrund ihres Akzents, ihres Aussehens oder ihres Namens [1, 5-7].

↑ nach oben

Menschen ohne Migrationshintergrund

Begriffskonstruktion zur Unterscheidung von Menschen mit Migrationshintergrund [1].

↑ nach oben

Migrant*in

Bei dem Begriff „Migrant*in“ handelt es sich um einen völkerrechtlich nicht definierten Oberbegriff für zugewanderte und abgewanderte Personen. Nach Definition des Bundesamtes für Bundesamt für Migration und Flüchtlinge (BAMF) umfasst der Begriff Personen, die im Ausland geboren wurden (und eine ausländische Staatsbürgerschaft haben) und nach Deutschland ziehen. Von einer synonymen Verwendung des Begriffes „Menschen mit Migrationshintergrund“ sollte abgesehen werden. Auch bei diesem Begriff gilt es zu berücksichtigen, dass es sich um keine homogene Gruppe handelt, sondern dass er Menschen mit verschiedenen biografischen Hintergründen aus unterschiedlichen Ländern umfasst [1, 8].

↑ nach oben

Moslem, Moslems

Veraltete und nicht mehr gebräuchliche Bezeichnung für Muslime, wird heutzutage zum Teil als Beschimpfung verwendet [1].

Vorschlag: Muslim, Muslima, Muslime

↑ nach oben

Muslim, Muslima, Muslime

Beschreibt Menschen mit Zugehörigkeit zur islamischen Religionsgemeinschaft. Häufig handelt es sich bei der Verwendung des Begriffs um eine Fremdzuschreibung, die auf äußerlichen Merkmalen beruht (→Antimuslimischer Rassismus) [1].

↑ nach oben

Person/People of Color (PoC)

Der Begriff wurde von Menschen, die sich in alltäglichen und/oder institutionellen Zusammenhängen Rassismus, Ausgrenzung und kollektiver Zuschreibung des „Nicht-weiß-seins“ ausgesetzt fühlen, als Selbstbezeichnung eingeführt. Gemeint ist damit nicht die Hautfarbe im biologischen Sinn. PoC kann diverse Menschen/Communitys mit verschiedenen biographischen Bezügen, z.B. Schwarze Deutsche, Sinti, Roma, Arabisch-Deutsche, Asiatisch-Deutsche umfassen.

Inzwischen wird häufiger von BPoC (Black and People of Color) gesprochen. Etwas seltener kommt hierzulande die Erweiterung BIPoC (Black, Indigenous and People of Color) vor, die explizit auch indigene Menschen mit einbeziehen soll. Diese Begriffe werden teils kritisiert, weil damit sehr große und unterschiedliche Gruppen vermengt werden [1].

↑ nach oben

Rom*nja, Roma

Bezeichnung der Angehörigen einer heterogenen Gruppe von Menschen (hinsichtlich Sprachen, Religionen und Gewohnheiten), die seit über 700 Jahren vorwiegend in Europa leben und die größte ethnische Minderheit Europas bilden, die vor über 1000 Jahren vermutlich aus dem indischen Subkontinent nach Europa ausgewandert ist [1].

Weiblich Singular: Romni, Weiblich Plural: Romnja; Männlich Singular: Rom; Männlich Plural: Roma

↑ nach oben

Schutzsuchende

Das Statistische Bundesamt definiert schutzsuchende Menschen wie folgt:

„Schutzsuchende sind Ausländerinnen und Ausländer, die sich unter Berufung auf völkerrechtliche, humanitäre oder politische Gründe in Deutschland aufhalten. Die Begründung für ihren Aufenthalt wird hierbei aus ihrem Aufenthaltsstatus im Ausländerzentralregister abgeleitet. Zu den Schutzsuchenden in Deutschland zählen die folgenden drei Kategorien von ausländischen Personen:

- Schutzsuchende mit offenem Schutzstatus halten sich zur Durchführung eines Asylverfahrens in Deutschland auf, wobei über ihren Schutzstatus noch nicht entschieden wurde.
- Schutzsuchende mit anerkanntem Schutzstatus besitzen einen befristeten oder unbefristeten Aufenthaltstitel aus dem humanitären Bereich des Aufenthaltsgesetzes.
- Schutzsuchende mit abgelehntem Schutzstatus halten sich nach Ablehnung im Asylverfahren oder nach Verlust ihres humanitären Aufenthaltstitels als Ausreisepflichtige in Deutschland auf [9].

Folgende Seite des Statistischen Bundesamtes bietet eine übersichtliche Grafik zum Thema Schutzstatus und Schutzsuchende:

https://www.destatis.de/DE/Themen/Gesellschaft-Umwelt/Bevoelkerung/Migration-Integration/Methoden/infografik-schutzsuchende-begriffe.pdf?__blob=publicationFile

↑ nach oben

Schwarze Deutsche

Nicht alle Deutschen sind *weiß*. Die Selbstbezeichnung Schwarze Deutsche ist von Schwarzen Rassismusforschenden gefordert und aktuell positiv empowernd besetzt. Der Begriff macht die gesellschaftliche Position der Menschen sichtbar, die von Rassismus betroffen sind. Schwarz wird bewusst großgeschrieben, da es sich nicht um die Hautfarbe handelt, sondern um ein durch die Gesellschaft konstruiertes Zuordnungsmuster [1, 8].

↑ nach oben

Schwer erreichbare Gruppen (hard-to-reach)

Bestimmte Personen oder Gruppen werden in den Bereichen Gesundheit oder Soziale Arbeit als „schwer erreichbar" oder „hard-to-reach“ beschrieben. Dazu gehören u.a. Menschen die Drogen konsumieren, HIV-Infizierte, Angehörige sexueller Minderheiten, Geflüchtete, Angehörige schwarzer und ethnischer Minderheiten und obdachlose Menschen. Bei der Nutzung des Begriffs ist zu berücksichtigen, dass es oft eine Reihe potenzieller Zugangsbarrieren zu Angeboten für Menschen gibt, wie z. B. schlechte Erfahrungen in der Vergangenheit, der Standort und die Öffnungszeiten der Angebote sowie die Art und Weise, wie Angebote finanziert und verwaltet werden. Die „schwere Erreichbarkeit“ ist somit nicht notwendigerweise ein Charakteristikum der Individuen, sondern eine Folge der (Fehl-)Gestaltung jeweiliger Systeme, die nicht auf eine angemessene Erreichung ausgelegt sind. Die Fremdbezeichnung kann von Personen der jeweiligen Gruppen als negativ konnotiert wahrgenommen werden. Darauf basierend gibt es Lösungsvorschläge, wie der Zugang zu Dienstleistungen für "schwer erreichbare" Personen und Gruppen verbessert werden kann, wie z.B. ein respektvoller Umgang, Aufbau von Vertrauen, Dienstleistungsflexibilität, partnerschaftliche Zusammenarbeit mit anderen Organisationen und Partizipation von Nutzenden [10].

↑ nach oben

Sinti*izze

Sinti*zze „ist die Bezeichnung für Nachfahren der Roma-Gruppen, die bereits im 14. und 15. Jh. in den deutschsprachigen Raum eingewandert sind. Sinti*zze sind die in West- und Mitteleuropa beheimateten Angehörigen der Minderheit. Die Bezeichnung wird jedoch nur in Deutschland, Österreich und Teilen Norditaliens verwendet. Außerhalb des deutschen Sprachraums wird Roma als Name für die gesamte Minderheit genutzt“ [1].

Weiblich Singular: Sintiza, Weiblich Plural: Sintize; Männlich Singular: Sintu; Männlich Plural: Sinti

↑ nach oben

Spätaussiedler*in

s. Aussiedler*in

↑ nach oben

Menschen mit Sprachproblemen (auch: keine oder schlechte Sprachkenntnisse, fehlende Sprachkenntnisse, schlechte Sprachfähigkeiten)

Mit Sprachproblemen wird im gesundheitswissenschaftlichen und medizinischen Bereich eine Reihe von logopädischen Erkrankungen und Störungen beschrieben [11].

Wenn im Kontext Migration von Menschen mit Sprachproblemen, keinen, schlechten oder fehlenden Sprachkenntnissen gesprochen wird, ist damit meistens gemeint, dass die Personen wenige oder keine Deutschkenntnisse haben. Es handelt sich also nicht um ein Sprachproblem oder fehlende Sprachkenntnisse, sondern um die Beschreibung der vorhandenen Kenntnisse der deutschen Sprache. Auch ist ein wertendes Adjektiv wie „schlecht“ ist keine adäquate Beschreibung von Sprachkenntnissen. Es sollte hierbei auch bedacht werden, dass viele Menschen mindestens eine, oft mehrere andere Sprachen sprechen [12].

Vorschlag: Menschen mit wenigen oder keinen Deutschkenntnissen

Türkischstämmig

Begriff ersetzt den Begriff „Türken“. Er berücksichtigt, dass viele aus der Türkei eingewanderte Menschen oder deren Nachkommen in der zweiten oder dritten Generation mittlerweile eine deutsche Staatsbürgerschaft haben. Da aber viele aus der Türkei eingewanderte Menschen Kurd*innen oder Angehörige anderer Minderheiten sind und sich nicht als „türkisch“ verstehen, wird mittlerweile die Bezeichnung „Türkeistämmig“ genutzt [1].

Vorschlag: Türkeistämmig

↑ nach oben

weiß

Mit *weiß* ist nicht die Schattierung der Haut eines Menschen gemeint, sondern eine gesellschaftspolitische Norm und Machtposition. Aufgrund dessen wird *weiß* in wissenschaftlichen Texten häufig klein und kursiv geschrieben. Mit *Weißsein* ist die privilegierte und dominante Position innerhalb des Machtverhältnisses Rassismus gemeint, die sonst unausgesprochen und unbenannt bleibt [1, 17].

↑ nach oben

Xenophobie

(griechisch xeno – fremd)

Form der gruppenbezogenen Menschenfeindlichkeit, bezeichnet die ablehnende Haltung gegenüber Personen/Gruppen, die als fremd wahrgenommen werden [5].

↑ nach oben

Zuwanderungsgesellschaft oder Aufnahmegesellschaft/ Aufnahmeland

Wird häufig für die Darstellung Deutschlands als Einwanderungsland verwendet. Sprachlich unterstreicht die Vorsilbe “zu“ eher die Nicht-Zugehörigkeit zu einer bestehenden Gemeinschaft [1]. Zuwanderungsgesellschaft betont die temporäre Begrenzung der Zugezogenen und unterscheidet sich damit von der Einwanderungsgesellschaft (siehe Einwanderungsgesellschaft) [18].

Vorschlag: Einwanderungsgesellschaft, Ankunftsland

↑ nach oben

Konzepte

Akkulturation

Ein u.a. in der Sozialepidemiologie angewandtes Konzept; zeigt viele Unklarheiten auf hinsichtlich der theoretischen Einbettung und Inkonsistenzen in der Operationalisierung und Messung. Generalisierungen und Zuschreibungen von Menschen zu einer „Kultur" sind auf Grund einzelner Merkmale kritisch zu hinterfragen. Der Begriff der Akkulturation als solcher sowie die Bildung eines Akkulturationsscores erscheinen nicht zielführend [13].

Vorschlag: Erhebung einzelner Dimensionen, wie z.B. Migrationsgeschichte, Sprachkenntnisse, subjektives Zugehörigkeitsgefühl und soziale Unterstützung können relevante Erklärungsfaktoren von Unterschieden der gesundheitlichen Lage sein.

↑ nach oben

Antidiskriminierung

Mit Antidiskriminierung verbindet sich ein aktives Eintreten gegen Diskriminierung, sei sie direkter oder indirekter Art. Die Spannweite von Aktivitäten reicht hierbei von der gezielten Beratung, dem Empowerment, der Unterstützung von Menschen mit Diskriminierungserfahrungen und der Dokumentation von Diskriminierungsfällen über öffentlichkeitswirksame Kampagnen für Vielfalt und gleiche Rechte bis hin zu Gesetzen mit einklagbaren Bestimmungen zum Schutz vor Diskriminierung. Ein wichtiges Ziel von Antidiskriminierungsarbeit ist die Sicherung fundamentaler Menschenrechte [4].

↑ nach oben

Antiziganismus

Antiziganismus wird verstanden als strukturelle Diskriminierung und als spezifische Form von Rassismus gegenüber Sinti*zze und Rom*nja. Der Begriff „Antiziganismus" bietet gegenüber Alternativen wie Antiromaismus oder Rassismus gegenüber Sinti*zze und Rom*nja Vorteile, denn er macht deutlich, dass die Betroffenengruppe nicht natürlich gegeben ist, sondern erst durch Diskriminierung sozial hergestellt wird [4]. Der stigmatisierende Begriff Z* ist dringend zu vermeiden.

↑ nach oben

Antiromaismus

Der von Romani-Aktivist*innen geprägte Begriff ist eine Alternative zum Begriff „Antiziganismus". Die Bezeichnung verwendet die Eigenbezeichnungen Rom*nja. Sie steht in der Kritik, eine „ethnisch" homogene Gruppe von Betroffenen vorzugeben. Die Tatsache, dass auch Personen und Gruppen, die sich nicht als Rom*nja identifizieren, von Antiromaismus betroffen sind widerspricht dieser [4].

↑ nach oben

Diskriminierung

Diskriminierung ist die ungleiche, benachteiligende und ausgrenzende Behandlung von gesellschaftlichen Gruppen und diesen zugeordneten Individuen ohne sachlich gerechtfertigten Grund. Diskriminierung kann sich zeigen als Kontaktvermeidung, Benachteiligung beim Zugang zu Gütern und Positionen, als Boykottierung oder als persönliche Herabsetzung. Der Begriff bezeichnet sowohl den Vorgang als auch das Ergebnis, also die Ausgrenzung und strukturelle Benachteiligung der diskriminierten Personen und Gruppen. Die Durchsetzung von Diskriminierung setzt in der Regel soziale, wirtschaftliche, politische oder diskursive Macht voraus. Diskriminierung ist nicht auf individuelles Handeln beschränkt, sondern auch in gesellschaftlichen, politischen, wirtschaftlichen und rechtlichen Strukturen verankert. Um dies deutlich zu machen, wird zwischen Diskriminierung auf subjektiver, interaktionaler, institutioneller, gesellschaftlich-kultureller und struktureller Ebene unterschieden [4].

↑ nach oben

Diversität

Der Begriff Diversität beschreibt die Vielfalt von Menschen und umfasst gruppenspezifische und individuelle Unterscheidungsmerkmale wie etwa ethnische Zugehörigkeit, soziale Herkunft, geschlechtliche Identität, sexuelle Orientierung, Behinderung, Alter, Religion [14].

↑ nach oben

Integration

Im Kontext der Migrationspolitik meint der Begriff oft „Assimilation“ (vollständige Anpassung) und impliziert eine individuelle Anpassungsleistung von Migrant*innen. Ein differenzierter Integrationsbegriff hingegen bezieht sich auf die gesamte Gesellschaft und deren Diversität und beschreibt eine chancengleiche Teilhabe aller an allen zentralen Bereichen des gesellschaftlichen Lebens [1, 15].

Vorschlag: Teilhabe, Partizipation, Chancengleichheit

↑ nach oben

Intersektionalität

Dieses Konzept wurde entwickelt, um bei der Analyse sozialer Ungleichheiten und Machtverhältnisse die Verschränkung und Wechselbeziehungen verschiedener sozialer Kategorien wie Geschlecht, Herkunft, Klasse usw. mitzudenken [16].

↑ nach oben

Kolonialismus

Als Kolonialismus wird die staatlich geförderte oder betriebene Besetzung eines Gebietes und die Fremdherrschaft über die dort ansässige Bevölkerung bezeichnet. Historisch lag die Hochzeit des Kolonialismus zwischen dem 15. und dem 20. Jahrhundert, als europäische (und später US-amerikanische und australische) Menschen begannen, Afrika, Teile Asiens und Amerika zu besiedeln und auszubeuten. Dabei unterdrückten, versklavten und töteten sie die lokale Bevölkerung und legitimierten dies mit einer rassistischen Ideologie, die ihre angebliche biologische, zivilisatorische und religiöse Überlegenheit behauptete. Auch das Deutsche Kaiserreich hatte mehrere Kolonien in Asien und Afrika. Bis in die 1970er Jahre hinein weigerten sich europäische Regierungen, den kolonisierten Gebieten ihre Unabhängigkeit zuzugestehen. Die Folgen des Kolonialismus sind noch heute spürbar – sowohl in den kolonisierten als auch ehemals kolonisierenden Gesellschaften [4].

↑ nach oben

Kultur

Kultur ist ein mehrdeutiger Begriff, der sich um die Gesamtheit der materiellen und immateriellen Ergebnisse menschlichen Wirkens dreht. Der enge Kulturbegriff bezieht sich auf Kunst und Geisteskultur, der weite auf die Lebenswelt des Menschen. Im Gegensatz zu klassischen Definitionen einer weitgehend homogenen,

statischen und in sich geschlossenen Kultur (Kulturalismus), die als Kollektiv verstanden wird, wird heute hervorgehoben, dass Kultur einem ständigen Veränderungsprozess unterliegt, in sich heterogen ist, nicht an ein bestimmtes Territorium gebunden und fließend ist. Menschen werden zudem als mehreren „Kulturen“ zugehörig betrachtet (z. B. Nationalität, Organisation, Religion, Generation, Familie), die durch zahlreiche Differenzlinien und mit ihnen verbundene Erfahrungen gebrochen sind. In diesem Sinne wird unter Kultur diejenige Lebenswelt verstanden, die eine Person als „eigene“ definiert, weil sie Normalität und Plausibilität bietet und soziales Routinehandeln ermöglicht [4].

↑ nach oben

Kulturalisierung

Unter Kulturalisierung wird die Praxis verstanden, Kultur als wesentliche, zentrale und determinierende Erklärung für (individuelle) Handlungen, Einstellungen, Verhaltensweisen, Konflikte oder Ausdrucksweisen zu verstehen. Häufig wird dabei der Kulturbegriff ethnisiert und Menschen werden beispielsweise auf ihre – angebliche – „türkische Kultur“ festgeschrieben. Dadurch werden sie in ihrer Vielfältigkeit und Komplexität nicht wahrgenommen, sondern ausschließlich auf eine (vermeintliche oder tatsächliche) kulturelle Zugehörigkeit reduziert. Dass es sich hierbei häufig um Fremdzuschreibungen und nicht um die eigene subjektive Identifikation handelt, gerät bei kulturalisierenden Interpretationen der Wirklichkeit häufig aus dem Blick. Durch Kulturalisierungen werden die Dichotomisierung (Zweiteilung) der Gesellschaft in Zugehörige („Wir“) und Nicht-Zugehörige („die Anderen“) verstärkt (Othering) und Stereotype und Zuschreibungen reproduziert [4].

↑ nach oben

Marginalisierte Bevölkerungsgruppen

Unter marginalisierten Bevölkerungsgruppen werden Menschen verstanden, die durch wirtschaftliche, soziale, geografische oder kulturelle Strukturen an den Rand der Gesellschaft verdrängt und somit marginalisiert sind. Marginalisierung geht mit einem Verlust von Ressourcen, Einflussmöglichkeiten und Status einher, kann auf unterschiedliche Weise erfolgen und unterschiedliche Bevölkerungsgruppen, u.a. auch Minderheitenpopulationen, betreffen.

Definition aus dem Papier des Robert Koch-Instituts Allgemeine Hinweise für Gesundheitsbehörden: Kontaktaufnahme und Zusammenarbeit mit marginalisierten Bevölkerungsgruppen während der COVID-19-Pandemie.

↑ nach oben

Othering

Das Konzept des Othering ist im Kontext der Postcolonial Studies entstanden und wurde u.a. durch Autor*innen wie Edward Said (1978) und Gayatri C. Spivak (1985) geprägt. Es stellt ein zentrales Moment postkolonialer Theoriebildung dar, dessen theoretische Ausarbeitung vor dem Hintergrund des Fortwirkens kolonialer Verhältnisse in heutigen Gesellschaften erfolgt. Es beruht auf der Unterscheidung in die Konstrukte „wir" und „Ihr". Das vermeintlich Andere wird dabei als Gegensatz zum hegemonialen Wir konstruiert und die Verschiedenheiten als hierarchisierend negativ bewertet. Die Konfrontation der Zuschreibung kann bei dem Gegenüber eine unbewusste Übernahme dieser Zuschreibung und somit zu einer Bestätigung des Prozesses des Otherings führen. Um das Eigen und die Norm des „wir" zu definieren, ist die Bestimmung der „Anderen" elementar [5, 9].

↑ nach oben

Parallelgesellschaft

Der Begriff wurde in den frühen 2000er Migrationsdebatten geprägt und beschreibt das vermeintliche Scheitern der Integration vorrangig türkischer Migrant*innen. Es wurde das Bild einer homogenen Minderheit konstruiert, die sich räumlich, sozial und kulturell von der Mehrheitsgesellschaft isoliert. Das Bild führte zu der Unterstellung einer Integrationsunwilligkeit unter Ausblendung eines gesamtgesellschaftlichen Beitrags zur gelungenen Integration. Andere Faktoren sollten hier mitgedacht werden, wie beispielsweise der Wohnungsmarkt als treibender Grund für die dichtere Ansiedlung von Einwander*innen in gewissen Stadtteilen [1].

↑ nach oben

Partizipation

Ist die aktive Einbeziehung von und partnerschaftliche Zusammenarbeit mit relevanten Akteur*innen oder Gruppen. Es werden spezifische Lebenswelten und -bedingungen berücksichtigt. Zielgruppen werden zu Akteur*innen und Betroffene zu Expert*innen. Partizipation führt zu Empowerment und nachhaltige und Adressat*innen-gerechte Lösungen.

↑ nach oben

Rasse/Race

Bei diesem Begriff handelt es sich um ein soziales Konstrukt, das benutzt wurde, um die europäische Herrschaft, Sklaverei, Vertreibung und Völkermord zu legitimieren. Es hat keine wissenschaftliche Grundlage. Es gibt keine menschlichen Rassen. Nach wie

vor wird der Begriff in Gesetzestexten (z.B. Art 3GG: „Niemand darf wegen seines Geschlechts, ... seiner Rasse ... benachteiligt oder bevorzugt werden.“) und in den Medien verwendet. Eine Entfernung des Begriffs aus den Gesetzestexten hat auf Länderebene teilweise bereits stattgefunden oder wird diskutiert [3, 5].

↑ nach oben

Rassismus

Rassismus ist der Prozess, in dem Menschen aufgrund tatsächlicher oder vermeintlicher Merkmale (z. B. Hautfarbe, Herkunft, Sprache, Religion) als homogene Gruppen konstruiert, hierarchisierend bewertet und ausgegrenzt werden. Der klassische Rassismus behauptet eine Ungleichheit und Ungleichwertigkeit von Menschengruppen auf Grundlage angeblicher biologischer Unterschiede. Im Neorassismus wird die Ungleichheit und Ungleichwertigkeit mit angeblichen Unterschieden zwischen „Kulturen“ zu begründen versucht. Rassismus kann seinen Ausdruck in Verhaltensweisen, Gesetzen, Bestimmungen und Anschauungen finden, die den Prozess der Hierarchisierung und Ausgrenzung unterstützen und gesellschaftliche Machtverhältnisse zur Grundlage haben und diese zugleich immer wieder reproduzieren. [4].

↑ nach oben

Hinweise zu problematischen Formulierungen

Begriffe mit Endungen wie „-strom/ströme“, „-schwemme“, „-influx“ oder „-welle“

Diese Begriffe umschreiben die Einreise von geflüchteten Menschen oder Migrant*innen mit Metaphern, die an unkontrollierbare Umweltkatastrophen und Bilder einer schutz- und hilflos ausgelieferten Bevölkerung erinnern. Durch solche Assoziationen werden Ängste und ein Bedarf an Sicherheits- und Schutzmaßnahmen suggeriert [1, 2, 17]. Darüber hinaus werden die Menschen hierdurch als Objekte bzw. als menschliche Masse, nicht als Subjekte konzipiert.

Vorschlag: erhöhte Wanderungsbewegungen oder Zuwanderung, transnationale Migration, Fluchtmigration

↑ nach oben

Begriffe mit der Endung „-krise“

Im Zusammenhang mit geflüchteten Menschen richtet dies fälschlicherweise den Fokus auf Geflüchtete als Verursachende einer vermeintlichen Krise [1, 2, 17].

Vorschlag: Herausforderungen, die sich aus der hohen Zahl an eingereisten geflüchteten Menschen ergeben

↑ nach oben

Kennzeichnung von Menschen als „illegal“

Menschen sollten nicht als „illegal“ bezeichnet werden. Nur Handlungen (oder auch ein Aufenthalt) können illegal oder gesetzeswidrig sein, Menschen nicht [1, 2, 17, 18].

Vorschlag: papierlose, illegalisierte, irreguläre, undokumentierte Menschen, Menschen ohne Papier, Sans-Papiers

Literatur

1. Neue Deutsche Medienmacher (NdM). *NdM-Glossar Wörterverzeichnis der Neuen deutschen Medienmacher*innen (NdM)mit Formulierungshilfen, Erläuterungen und alternativen Begriffen für die Berichterstattung in der Einwanderungsgesellschaft*. 2021 [cited 2021 09.04.2021]; Available from: glossar.neuemedienmacher.de.
2. Caritas Frankfurt, *Flucht und Migration: Empfehlung für einen sensiblen Sprachgebrauch.* 2016.
3. AntiDiskriminierungsBüro (ADB) Köln / Öffentlichkeit gegen Gewalt e.V., ed. *Sprache schafft Wirklichkeit. Glossar und Checkliste zum Leitfaden für einen rassismuskritischen Sprachgebrauch*. 2019.
4. Informations- und Dokumentationszentrum für Antirassismusarbeit e. V. (IDA). *Glossar*. 06.10.2021]; Available from: https://www.idaev.de/recherchetools/glossar/.
5. Bednaschewsky, R. and L. Supik, *Vielfältig Deutschsein – Von Deutschen of Color und Deutschen mit Migrationshintergrund in der Statistik*, in *Rassismus und Rechtsextremismus in Deutschland - Figurationen und Interventionen in Gesellschaft und staatlichen Institutionen,*, M. Gomolla, M. Menk, and E. Kollender, Editors. 2018, Beltz-Juventa. p. 179-194.
6. DESTATIS. *Personen mit Migrationshintergrund*. [cited 2020 23.09.2020]; Available from: https://www.destatis.de/DE/Themen/Gesellschaft-Umwelt/Bevoelkerung/Migration-Integration/Methoden/Erlauterungen/migrationshintergrund.html.
7. Will, A.K., *https://rat-fuer-migration.de/2022/06/07/rfm-debatte-2022/*. 2022.
8. Amadeu Antonio Stiftung. *Antisemitismus- und rassismuskritische Jugendarbeit. Ein Glossar.* . 2014 [cited 2020 23.09.2020]; Available from: https://www.amadeu-antonio-stiftung.de/publikationen/glossar-antisemitismus-und-rassismuskritische-jugendarbeit/.
9. DESTATIS, *Bevölkerung und Erwerbstätigkeit. Schutzsuchende. Ergebnisse des Ausländerzentralregisters.* Fachserie 1 Reihe 2.4, 2021.

10. Flanagan, S.M. and B. Hancock, *'Reaching the hard to reach'--lessons learned from the VCS (voluntary and community Sector). A qualitative study.* BMC Health Serv Res, 2010. **10**: p. 92.
11. *Sprachtherapie: Nicht zu früh.* Dtsch Arztebl International, 2000. **97**(38): p. A-2417.
12. Geis-Thöne, W., *IW-Trends 1/2022. Kinder mit nicht deutschsprechenden Eltern*, in *Vierteljahresschrift zur empirischen Wirtschaftsforschung*, I.d.d.W.K.e. V, Editor. 2022: Köln.
13. Schumann, M., et al., *Konzepte für ein migrationssensibles Gesundheitsmonitoring.* Journal of Health Monitoring 2019. **4**(3).
14. Albert-Ludwigs-Universität Freiburg. *Diversity-Glossar*. 2021 [cited 2022 05.07.2022]; Available from: https://www.diversity.uni-freiburg.de/bereich-gender-und-diversity/Lehre/210111_Glossar_web.pdf.
15. Deutscher Caritasverband e.V. *Fact Sheet Begriffsklärung Integration und Inklusion*. 2018 06.10.2021; Available from: https://www.dbk.de/fileadmin/redaktion/microsites/fluechtlingshilfe/Caritas_Factsheet_Begriffsklaerung_Integration_Inklusion.pdf.
16. Walgenbach, K. *Intersektionalität – eine Einführung*. 2012 06.10.2021]; Available from: www.portal-intersektionalität.de.
17. Grove, N.J. and A.B. Zwi, *Our health and theirs: forced migration, othering, and public health.* Soc Sci Med, 2006. **62**(8): p. 1931-42.
18. International Organization for Migrantion, *International Migration Law: Glossary on Migration (Vol. 34)*. 2019, International Organization for Migrantion: Geneva.
